# Novel role of O-glycosyltransferases GALNT3 and B3GNT3 in the self-renewal of pancreatic cancer stem cells

**DOI:** 10.1186/s12885-018-5074-2

**Published:** 2018-11-22

**Authors:** Srikanth Barkeer, Seema Chugh, Saswati Karmakar, Garima Kaushik, Sanchita Rauth, Satyanarayana Rachagani, Surinder K. Batra, Moorthy P. Ponnusamy

**Affiliations:** 1 Department of Biochemistry and Molecular Biology, University of Nebraska Medical Center, Omaha, NE 68198-5870 USA; 2 Fred and Pamela Buffett Cancer Center, University of Nebraska Medical Center, Omaha, NE 68198 USA

**Keywords:** Pancreatic cancer stem cells, Glycosylation, Tunicamycin, GALNT3, B3GNT3, CD44v6

## Abstract

**Background:**

Glycosylation plays a critical role in the aggressiveness of pancreatic cancer (PC). Emerging evidences indicate significant involvement of cancer stem cells (CSCs) in PC aggressiveness. However, the importance of glycosylation in pancreatic cancer stem cells (PCSCs) is yet to be addressed. Hence, we evaluated the potential role of glycosylation in maintenance of stemness of PCSCs.

**Methods:**

Effect of glycosylation specific inhibitors on growth and PCSCs of PC cells was assessed by MTT assay and Side Population (SP) analysis. Isolated PCSCs/SP were characterized using molecular and functional assays. Expression of tumor-associated carbohydrate antigens (TACAs) was analyzed in PCSCs by western blotting. Effect of tunicamycin on PCSCs was analyzed by tumorsphere, clonogenicity, migration assay and immunoblotting for CSCs markers. The differential expression of glycogenes in PCSCs compared to non-CSCs were determined by RT-qPCR, immunoblotting and immunofluorescence. Co-expression of GALNT3 and B3GNT3 with CD44v6 was assessed in progression stages of *Kras*^*G12D*^*; Pdx-1-Cre* (KC) and *Kras*^*G12D*^*; p53*^*R172H*^*; Pdx-1-Cre* (KPC) tumors by immunofluorescence. Transient and CRISPR/Cas9 silencing of GALNT3 and B3GNT3 was performed to examine their effect on CSCs maintenance.

**Results:**

Inhibition of glycosylation decreased growth and CSCs/SP in PC cells. PCSCs overexpressed CSC markers (CD44v6, ESA, SOX2, SOX9 and ABCG2), exhibited global expressional variation of TACAs and showed higher self-renewal potential. Specifically, *N*-glycosylation inhibition, significantly decreased tumorsphere formation, migration, and clonogenicity of PCSCs, as well as hypo-glycosylated CD44v6 and ESA. Of note, glycosyltransferases (GFs), GALNT3 and B3GNT3, were significantly overexpressed in PCSCs and co-expressed with CD44v6 at advanced PDAC stages in KC and KPC tumors. Further, GALNT3 and B3GNT3 knockdown led to a decrease in the expression of cell surface markers (CD44v6 and ESA) and self-renewal markers (SOX2 and OCT3/4) in PCSCs. Interestingly, CD44v6 was modified with sialyl Lewis a in PCSCs. Finally, CRISPR/Cas9-mediated GALNT3 KO significantly decreased self-renewal, clonogenicity, and migratory capacity in PCSCs.

**Conclusions:**

Taken together, for the first time, our study showed the importance of glycosylation in mediating growth, stemness, and maintenance of PCSCs. These results indicate that elevated GALNT3 and B3GNT3 expression in PCSCs regulate stemness through modulating CSC markers.

**Electronic supplementary material:**

The online version of this article (10.1186/s12885-018-5074-2) contains supplementary material, which is available to authorized users.

## Background

Pancreatic cancer is a highly lethal malignancy, estimated to become the second-leading cause of cancer related deaths by 2030 [1]. Early metastasis and increased chemoresistance make this cancer extremely difficult to treat and results in worse than typical prognosis [2, 3]. Emerging evidences support the involvement of CSCs in metastasis and resistance to chemotherapy [4, 5]. CSCs are a rare, small subset of cells with the capacity to give rise to full tumor mass. They have a self-renewal capacity and undergo asymmetric or symmetric cell division producing a heterogeneous cell population [5–7]. The presence of CSCs has been observed in many solid tumors, including PC [8–10]. PCSCs have been identified by expression of markers such as CD44^+^CD24^+^ESA^+^, CD133^+^, ALDH1^+^ and Side population (SP, Hoechst dye exclusion) [8]. CD133^+^CXCR4^+^ was identified as a subpopulation of PCSCs that mediates tumor metastasis in PC [8, 10, 11].

In recent years, various reports have shown the role of glycosylation in tumorigenesis. Glycosylation is a co- or post-translational modification of proteins and lipids with glycans (Glycoproteins; *N*- or *O*-linked, O-GlcNAc and Glycolipids). Glycosylation regulates various biological and cellular signaling, including embryogenesis, pluripotency, cell-to-cell & cell-to-environment interaction, signal transduction, protein folding, defense against microbial infection, and immune modulation [12–14]. Aberrant glycosylation is associated with tumor initiation, development, and metastasis. The expression of TACAs like Tn, sTn, T, sT (truncated) and sialyl Lewis a (sLe^a^), sialyl Lewis x (sLe^x^) (de novo; neo-synthesis) are especially implicated in tumor formation and metastasis in many cancers, including PC [15–17].

Several studies have explored the role of *O*-linked glycosylation in the regulation of tumorigenesis in PC. Expression of truncated *O*-glycans induced oncogenic signaling in PC; however, expression of core-3 derived *O*-glycans (extended structures) was shown to inhibit tumor growth and metastasis [18, 19]. In our recent study, we demonstrated that the crossing of C1galt1 floxed mice  with *Kras*^*G12D*^*; p53*^*R172H*^*; Pdx-1-Cre* mice resulted in elevated synthesis of truncated *O*-linked glycans promoting development of aggressive PDAC with increased metastasis [17]. De-regulated mucin expression (MUC1, MUC4, MUC5AC, MUC16; majorly *O*-linked glycoprotein) has been implicated in PC development, metastasis, and chemoresistance [16, 20–24]. However, there are limited studies on the role of glycosylation in stemness of PCSCs [25]. One study, for example, demonstrated the enrichment of fucosylation in gemcitabine-resistant and CD44^+^CD24^+^ CSC-like populations in PC [26]. In another, the glycosyltransferase ST6Gal-1 was shown to regulate stem cell transcription factors, confer CSC phenotype, and promote gemcitabine resistance in pancreatic and ovarian cancer [27]. However, no detailed study has been performed to understand the role of glycosylation in stemness of PCSCs.

Given that both CSC and altered glycosylation have been implicated in tumorigenesis, metastasis, and chemoresistance, we tested the hypothesis that glycosylation maintains the stemness in PCSCs. The present study was undertaken to elucidate the significance of glycosylation and to identify the glycosyltransferases involved in the maintenance of stemness of PCSCs, and our results demonstrated significant importance of glycosylation in stemness. We also defined the role of specific GFs in the maintenance of stemness in PCSCs.

## Methods

### Cell culture

SW1990 (Cat. No. CRL-2172) and Capan1 (Cat. No. HTB-79) cells were procured from American type Culture Collection (Manassas, VA, USA) and cultured in Dulbecco’s Modified Eagles Medium (DMEM) (HyClone Laboratories, Logan, UT, USA) supplemented with 10% fetal bovine serum, 2 mM glutamine and 1% penicillin-streptomycin solution (Sigma-Aldrich, St Louis, MO, USA). These cell lines were recently authenticated by STR confirmation (Nebraska Medical Center HDI Lab, UNMC, Omaha, NE) and checked for mycoplasma contamination by using Roche BM cyclin kit (Cat. No. 10799050001) according to manufacturer’s instruction (Roche, Peznberg, Germany).

### Growth inhibitory studies

Cells seeded at 4 × 10^3^ cells/per well density in 96 well plate and grown in complete DMEM for 24 h. Cells were treated with different concentration of tunicamycin ™ (5–20 μg/ml) and benzyl-2-acetamido-2-deoxy-α-D-galactopyranoside (BAG) (0.5–2 mM) for different time points (24-72 h). At specified time points, 10 μl of MTT (5 mg/ml) in plain DMEM (90 μl) added to the each well and incubated for 3–4 h. Finally, cells were lysed by adding 200 μl DMSO to each well and absorbance was measured at 570 nm, with reference wavelength of 640 nm using ELISA plate reader.

### SP assay

We performed SP analysis to determine the effect of TM (1.2 μM) and BAG (0.5 mM) on PCSCs. SP analysis was carried out by staining cells with Hoechst 33342 dye (AnaSpec Inc., Fremont, CA, USA) as described earlier [28]. Verapamil/Reserpine, a calcium channel blocker, was used as a control for gating SP cells.

### Isolation of SP/CSC population from PC cells and culture

SP cells or putative CSCs and non-side population were isolated from SW1990 and Capan1 cells by FACS (fluorescent activated cell sorting) using standard method [28]. Verapamil/Reserpine was used to gate and sort SP cells. Isolated SP from SW1990 and Capan1 were grown in standardized stem cell-specific media as described previously [29]. SP cells were grown in 0.1 μM gemcitabine to obtain an enriched CSC population.

### Immunoblot and lectin blot assay

SW1990, Capan1, SW1990 SP and Capan1 SP processed for protein extraction and western blotting using standard procedures [30]. For protein detection, the following antibodies were used; anti-OCT3/4, anti-SOX2, anti-ESA, anti-CD24, anti-β-Actin; anti-GAPDH (Sanatacruz Biotechnology, TX, USA); anti-CD44S (Cell signaling technology, MA, USA); anti-CD44v6 (eBiosciences, CA, USA); anti-CD133 (Abnova, CA, USA); anti-SOX9, anti-GALNT3, anti-B3GNT3, anti-MGAT4A (Abcam, MA, USA). Anti-CC49, Anti-sLe^x^ (Additional file 1: Table S1); Anti-sLe^a^ (Thermo Fischer Scientific, USA). β-Actin /GAPDH was used as a loading control. Blots were developed using chemiluminescent HRP kit (Bio-Rad, CA, USA). For lectin blotting, blots were blocked by 3% BSA (Jackson Immunoresearch Labs, Inc.) and probed for biotinylated lectins including VVA (binds Tn antigen) and PNA (binds T antigen) (Vector Labs. CA, USA). Streptavidin HRP was applied and bands were visualized using a chemiluminescent HRP kit.

### Tumorsphere assay

NSP and SP cells of SW1990 and Capan1 were seeded in a 24/96 well ultra-low attachment plate (Corning Inc., New York, USA) in CSC-specific media at a concentration of 500/2500 cells per well in triplicate. Tumorsphere formation was observed under the microscope after 7–14 days. The number of tumor spheres were counted and plotted. A similar procedure was carried out for TM-treated (0.6 and 1.2 μM) and CRISPR knockout (KO) cells to study tumorsphere formation with respective controls.

### RNA isolation and real time-quantitative polymerase chain reaction (RT-qPCR)

Total RNA was isolated from cells using RNAeasy kit (Qiagen, CA, USA). 1–2 μg of RNA from each sample was converted to cDNA and used for RT-qPCR analysis [31]. Expression of CSC genes and glycogenes was profiled using gene-specific primers (Additional file 1: Table S2) and relative expression calculated using 2^−ΔΔCT^ method. Data expressed as mean ± s.e.m. and statistical significance was set at *P ≤* 0.05.

### Human glycosylation polymerase chain reaction (PCR) array

Total RNA isolated from NSP and SP cells of SW1990 were reverse-transcribed using RT^2^ SYBR qPCR master mix (330,401, Qiagen). 25 μl aliquot of mix of both samples was added in separate 96-well PCR array kit containing lyophilized gene-specific primer set (PAHS-046ZF-12, Qiagen). Threshold cycles were used to calculate fold change using free online web server of RT^2^ profiler PCR array data analysis [31].

### Confocal immunofluorescence (IF) microscopy

Cells were grown on sterilized cover slips for 24 h and primary antibodies specific for mouse-CD44v6 (1:500), rabbit-ESA (1:200), rabbit-SOX2 (1:50), rabbit-GALNT3 (1:200), mouse-GALNT3 (1:20), rabbit-B3GNT3 (1:200) were used to stain cells overnight at 4 °C. After incubation, cells were processed using standard procedure [29]. We used KC and KPC spontaneous mouse models and their control littermate tissue sections to examine GFs expression in CSCs. KC and KPC tissue sections were stained with mouse-CD44v6 (1:100), rabbit-GALNT3 (1:50), and rabbit-B3GNT3 (1:200) overnight at 4 °C. After incubation, tissues were processed as described earlier [29, 32].

### Colony forming assay

Cells treated with or without TM (0.6 and 1.2 μM) were seeded in triplicate in six well plates at a concentration of 500/1000 cells per well in CSC-specific media. After 10–14 days of seeding, cells were fixed with ice-cold methanol for 5 min and stained with crystal violet stain solution (0.1%, *w*/*v* in 20 nM 4-morpholinepropanesulfonic acid; Sigma, MO, USA). Colonies were counted manually or with ImageJ software. Similar procedure was carried out for CRISPR knockout cells to study colony formation with controls.

### Trans well migration assay

SP cells treated with or without TM (0.6 and 1.2 μM) were seeded at a concentration of 0.1 million cells per well in 100 μl of plain medium into the upper well of Boyden chamber with 8-μm pore, 6.5 mm polycarbonate trans well filters (Corning Costar Corp., MA, USA). The lower chamber contained 600 μl of CSC media. After 24 h, cells that had migrated at the lower surface of the membrane were fixed, stained, and counted under microscope. Similar procedure followed to study migration upon GALNT3KO clones.

### Knockdown (KD) of GALNT3 and B3GNT3

Transient KD of GALNT3 (Cat. No. SR301729) and B3GNT3 (Cat. No. SR307003) was performed in SP cells of SW1990 and Capan1 cells using gene-specific siRNA (Origene, MD, USA). SP cells of SW1990 and Capan1 were plated 0.2 million cells/well in a six-well plate. Next day, cells were serum-starved for 3–4 h and transfected with gene-specific SiRNA or control siRNA at a concentration of 80 nM/well by using lipofectamine 2000 reagent (Invitrogen, Life Technologies Inc., NY, USA) in plain DMEM. After 4–6 h of transfection, serum containing media was added and cell lysate collected at 48–72 h post transfection.

### Immunoprecipitation (IP) analysis

Cells were lysed with IP buffer (20 mM Tris, pH 7.5, 200 mM NACL, 1% NP-40, 10% Glycerol, 1 mM DTT, Protease inhibitor cocktail). IP was performed with anti-CD44v6, anti-CD44S, and anti-SLe^a^ (Thermo Fischer, MA, USA) antibodies as described earlier [30]. Immunoprecipitated samples were resolved on 10% SDS-PAGE (sodium dodecyl sulfate polyacrylamide gel electrophoresis) and transferred on PVDF (Polyvinylidene difluoride) membrane. Blots were incubated with protein-specific primary antibody, followed by species-specific secondary antibody and developed with chemiluminescent kit.

### CRISPR/Cas9-mediated KO

KO of GALNT3 was performed by using CRISPR/Cas9 system in Capan1 SP cells. Briefly, cells were transfected with GALNT3 guide RNA (5’ATTTCTTTGCACCGAGATCT3’, GenScript, NJ, USA) in CRISPR/Cas9 vector (pSpCas9 BB-2A-GFP (PX458), GenScript, NJ, USA) by using lipofectamine 2000 reagent. GFP positive single cells were sorted in 96 well plate after 48 h of transfection by FACS. Single cells were allowed to grow into colonies in CSC-specific media and later used for further analysis.

### Statistical analysis

Different statistical analysis including student t-test, one-way, and two-way Annova were used for different experiments to determine statistical significance. Error bars were set calculate standard error values. *P* value of ≥0.05 was considered statistically significant. * P ≤ 0.05 ** P ≤ 0.01 *** P ≤ 0.001 **** P ≤ 0.0001.

## Results

### Inhibition of glycosylation reduces the SP of PC cells

**Fig. 1:**
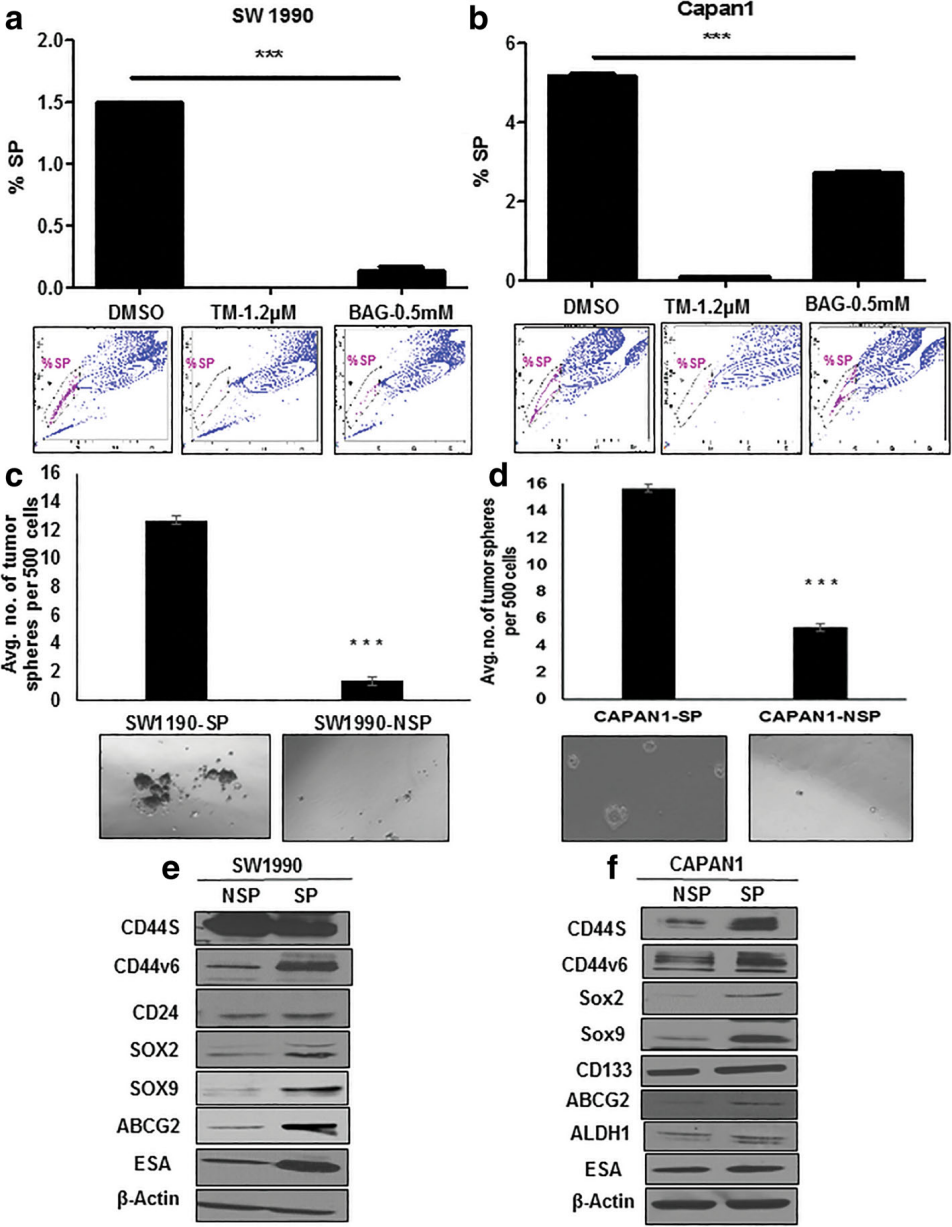
Inhibition of global glycosylation reduces the SP cells of PC. **a** and **b** SP analysis in SW1990 and Capan 1 cells after treatment with TM and BAG for 48 h, respectively. Reserpine used as control to gate the SP cells. **c** and **d** Tumorsphere formation assay from NSP and SP cells of SW1990 and Capan1, respectively. **e** and **f** Immunoblotting analysis of CSC marker expression from NSP and SP cells of SW1990 and Capan1, respectively

To investigate the functional importance of glycosylation in the stemness of PC cells, we used glycan inhibitors and analyzed a CSC population. TM and BAG were used to inhibit *N*-linked and *O*-linked glycosylation, respectively. Both TM and BAG showed dose- and time-dependent growth inhibition of SW1990 and Capan1 cells (Additional file 2: Figure S1a-S1d). We further analyzed the SP/CSCs and altered glycosylation with TM and BAG treated SW1990 and Capan1 cells. Both TM and BAG significantly reduced the SP/CSC population in SW1990 and Capan1 cells, as determined by SP analysis (Fig. 1a and b). TM treatment resulted in altered *N*-linked glycosylation of stem cell markers (ESA and CDDv6) and BAG treatment resulted in global variation of Tn antigen, *O*-linked glycosylation, as detected by VVA staining in PC cells (Additional file 2: Figure S1e and S1f).

### Cancer stem cell-like phenotype of isolated SP cells

**Fig. 2:**
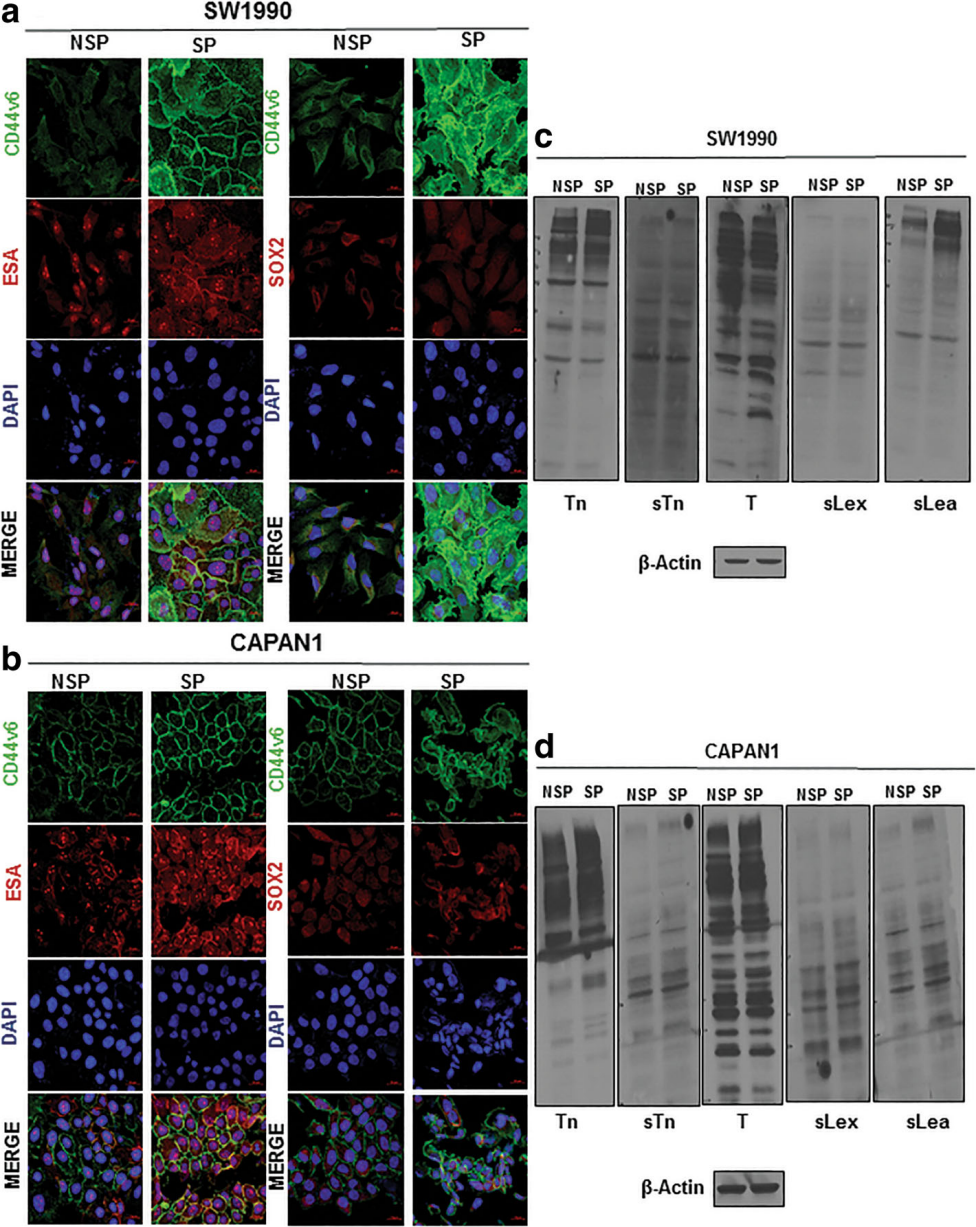
Global variation in expression of TACAs in PCSCs. **a** and **b** IF analysis of CSC marker expression from NSP and SP cells of SW1990 and Capan1, respectively. **c** and **d** Immunoblotting analysis of TACAs from NSP and SP cells of SW1990 and Capan1, respectively

Because PCSCs display elevated expression of CSC markers and pose a marked self-renewal property, we characterized the SP of SW1990 and Capan1 cells [28]. We isolated SP cells and non-side population (NSP) cells from SW1990 and Capan1 cells (Additional file 3: Figure S2a), and found that the cellular morphology of SP cells was distinct from that of NSP cells derived from either SW1990 or Capan1 (Additional file 3: Figure S2b). Furthermore, the CSC phenotype of SP cells were characterized by tumorsphere assay and CSC markers expression. Tumorsphere assay determines the self-renewal capacity and in vitro tumorigenic potential of stem cells [33]. SP cells from SW1900 and Capan1 formed a significantly higher number of tumorspheres compared to NSP cells (Fig. 1c and d). Further, SP cells showed increased expression of cell surface markers (viz CD44v6, CD44s, ESA, and ABCG2) and self-renewal markers (viz SOX2 and SOX9) compared to NSP as determined by RT-qPCR (Additional file 3: Figure S2c and S2d) and western blotting (Fig. 1e and f). Increased expression of CSCs markers in SP cells (CD44v6 with SOX2/ESA) was also confirmed by IF analysis (Fig. 2a and b).

### TACAs differentially expressed in PCSCs

Aberrant glycosylation in cancer results in the expression of TACAs that are implicated in early tumor progression and metastasis [15]. Because CSCs have been implicated in tumor initiation and metastasis, we next examined if expression of TACAs, such as Tn, T, sTn, sLe^x^, and sLe^a^, is altered in CSCs/SP cells of SW1990 and Capan1 PC cells. SP cells of SW1990 and Capan1 showed differential expression of TACAs compared to NSP (Fig. 2c and d). Few higher and low molecular bands of Tn and sLe^a^ exhibited greater expression in SP cells of SW1990 and Capan1 compared to NSP cells (Fig. 2c and d).

### *N*-linked glycosylation regulates stemness, clonogenicity, migration of PCSCs

**Fig. 3:**
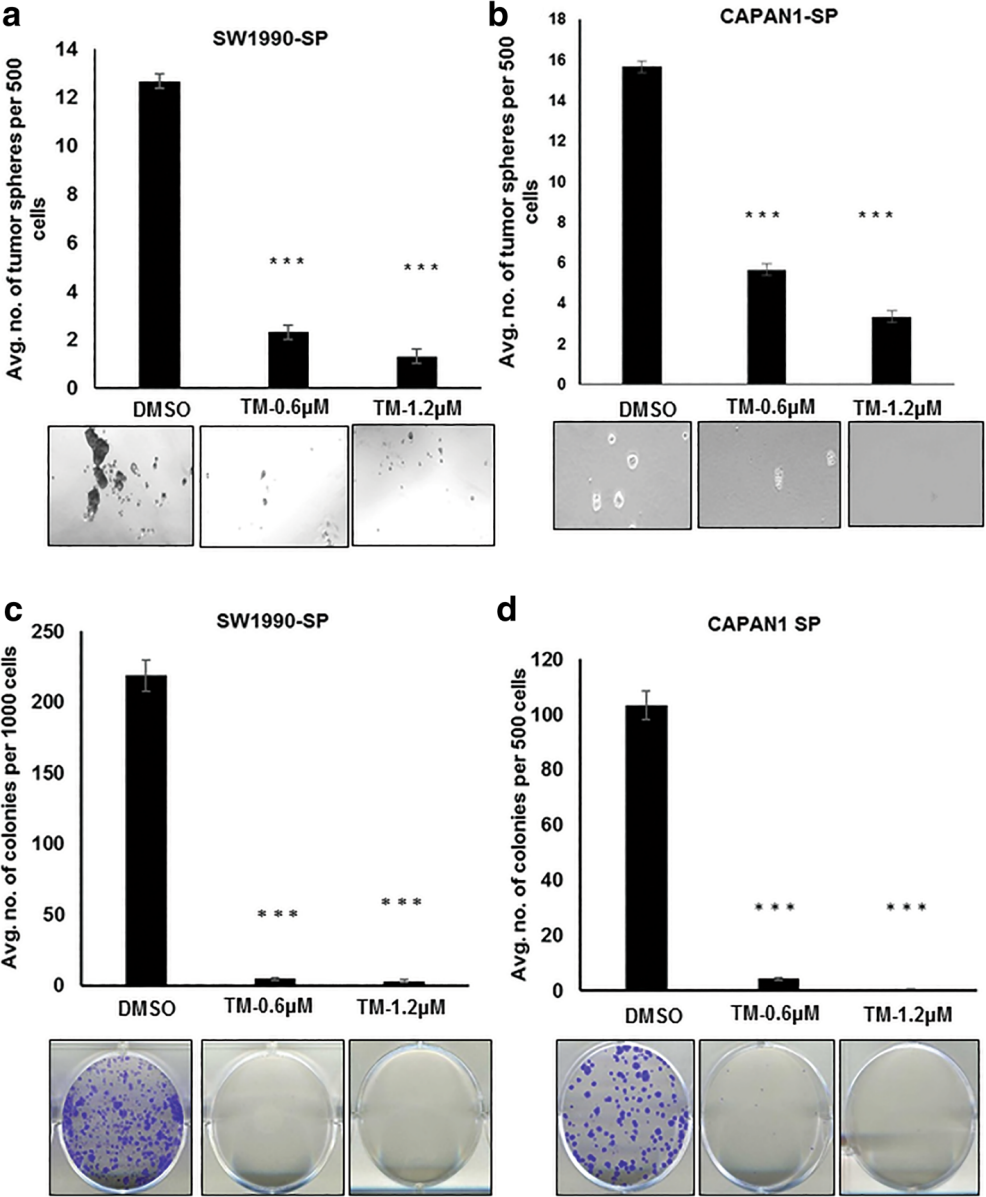
TM inhibits tumorsphere formation and clonogenicity of PCSCs. **a** and **b** Tumorsphere formation assay from SP cells of SW1990 and Capan1, respectively, after pre-treatment with TM for 48 h. **c** and **d** Colony formation assay from SP cells of SW1990 and Capan1, respectively, after pre-treatment with TM for 48 h

**Fig. 4:**
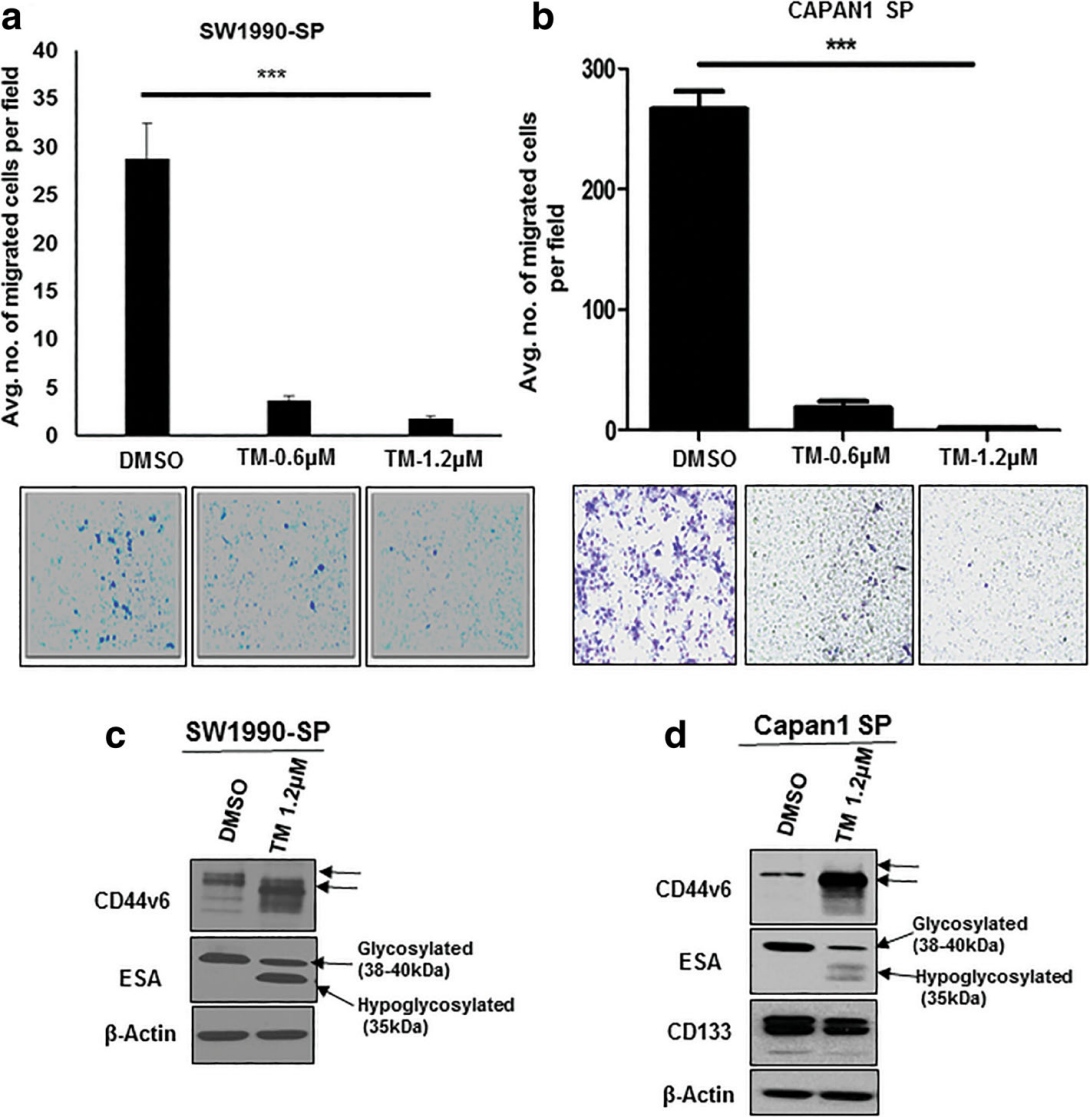
TM inhibits migration of PCSCs and hypo-glycosylates CSC markers. **a** and **b** Trans-well migration assay from SP cells of SW1990 and Capan1, respectively, after treatment with TM. **c** and **d** Immunoblotting analysis of CSC marker expression from SP cells of SW1990 and Capan1, respectively, after treatment with TM for 48 h

Because many of the CSC markers, such as CD44 and ESA, are *N*-glycosylated, we evaluated the role of *N*-linked glycosylation in the stemness of PCSCs by treating them with TM [34, 35]. TM treatment significantly inhibited the tumorsphere formation of SP cells of SW1990 and Capan1 compared to control cells treated with dimethyl sulfoxide (DMSO) (Fig. 3a and b). Further, experiments were performed to understand the role of *N*-linked glycosylation in growth and migration of PCSCs. Of note, TM treatment resulted in a complete loss of ability of SP cells from SW1990 and Capan1 to form visible colonies, compared to DMSO-treated cells (Fig. 3c and d). In addition, TM treatment significantly inhibited the migratory capacity of SW1990 and Capan1 SP cells compared to DMSO treatment (Fig. 4a and b). These results suggest the importance of *N*-linked glycans in stemness, clonogenicity and migration. We further examined TM effect on CSC marker expression, and were interested to find that TM treatment resulted in a shift in molecular weight of CD44v6 and ESA in SP cells of SW1990 and Capan1 compared to DMSO treatment, and resulted in a reduction of expression of CD133 in Capan1 SP cells (Fig. 4c and d). This observation indicates the possible involvement of *N*-linked glycan modification of CD44v6 and ESA in PCSC stemness, tumorigencity, and metastasis.

### GALNT3 and B3GNT3 overexpressed in PCSCs

**Fig. 5:**
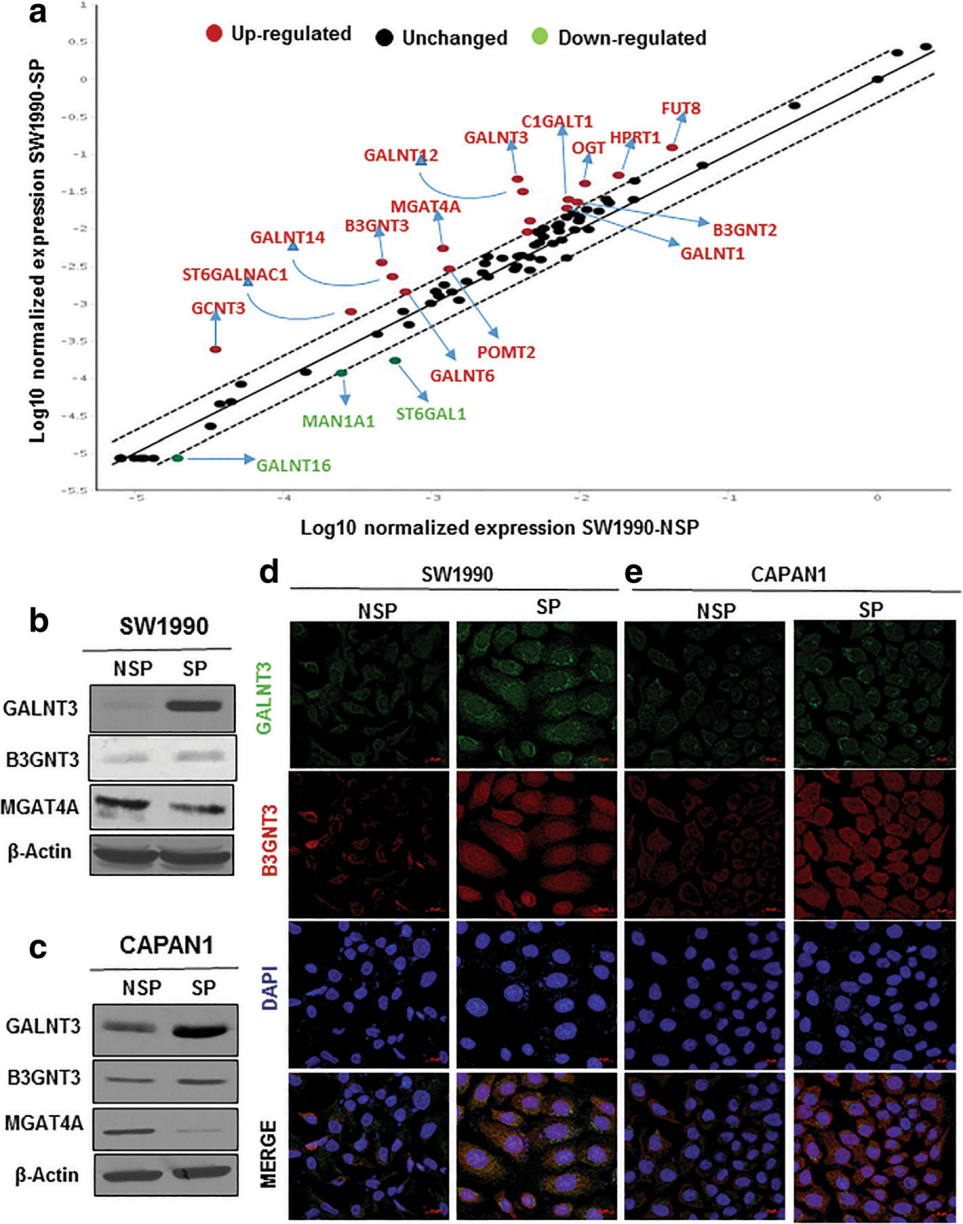
GALNT3 and B3GNT3 are overexpressed in PCSCs. **a** PCR array for human glycosylation from NSP and SP cells of SW1990. **b** and **c** Immunoblotting analysis of DEGs between NSP and SP cells of SW1990 and Capan 1, respectively. **d** and **e** IF analysis of GALNT3 and B3GNT3 expression between NSP and SP cells of SW1990 and Capan 1, respectively

Since aberrant glycosylation is attributed to altered expression of GFs, we next performed a PCR array for human glycosylation genes in SW1990 NSP and SP cells. Twenty glycogenes were differentially expressed from PCR array for human glycosylation between NSP and SP cells of SW1990 (Fig. 5a, Additional file 1: Table S3). Differentially expressed glycogenes (DEGs) were further validated using RT-qPCR, revealing that only GALNT3, GALNT12, GALNT16, B3GNT2, B3GNT3, and MGAT4A were differentially expressed (Additional file 4: Figure S3a, Additional file 1: Table S3). These DEGs were also validated in NSP and SP of Capan1 by RT-qPCR (Additional file 4: Figure S3b). In particular, increased expression of GALNT3 and B3GNT3 and decreased expression of MGAT4A proteins was observed in SP cells from SW1990 and Capan1 cells (Fig. 5b and c). Increased expression of GALNT3 and B3GNT3 was seen in both the mRNA and protein levels, whereas MGAT4A expression alone was less in protein level. The IF expression of GALNT3 and B3GNT3 confirmed overexpression of these GFs in PCSCs (Fig. 5d and e).

### GALNT3 and B3GNT3 specifically over-expressed in advanced stages of KC and KPC autochthonous tumors along with CD44v6

**Fig. 6:**
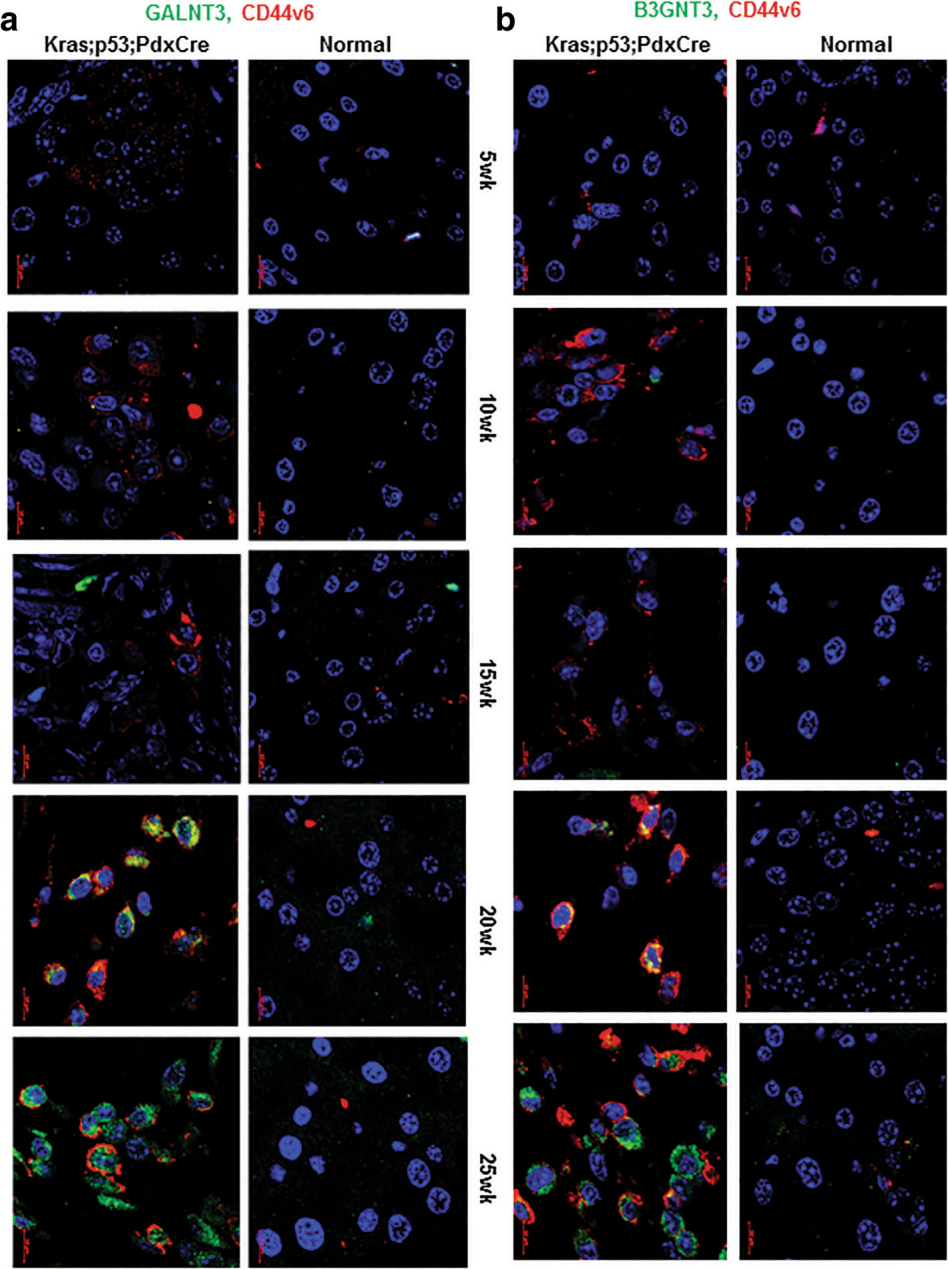
GALNT3 and B3GNT3 expresses at PDAC stage in PCSCs of KPC. **a** and **b** IF analysis for co-expression of GALNT3 and B3GNT3 with CD44v6, respectively, at different stages of KPC

To understand the involvement of GALNT3 and B3GNT3 in the pathogenesis of PC progression, the expression of these GFs, along with CSC marker CD44v6, were analyzed in different stages of KC and KPC mouse tumor tissues by IF. In KC tissues, GALNT3 and B3GNT3 co-expression with CD44v6 were observed at fully advanced PDAC stage at the 50th week (Additional file 5: Figure S4a and S4b). Similarly, in KPC tissues, co-expression of GALNT3 and B3GNT3 with CD44v6 observed at PDAC stages at the 20th and 25th weeks (Fig. 6a and b). These results indicate that both GALNT3 and B3GNT3 are specifically overexpressed in advanced stages of PC progression, along with CSC marker CD44v6 expression.

### KD of GALNT3 and B3GNT3 decreases PCSC markers

**Fig. 7:**
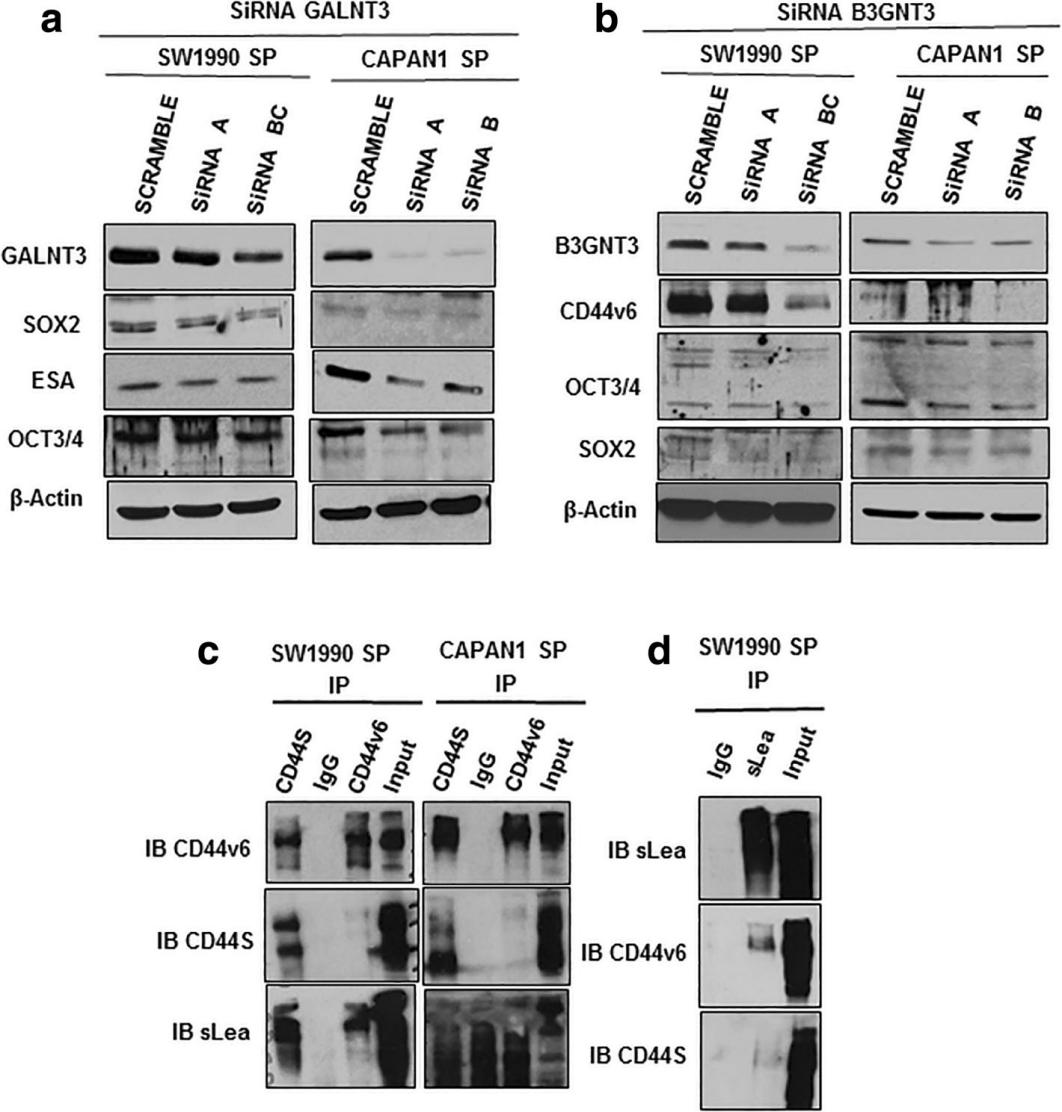
GALNT3 and B3GNT3 regulates expression of PCSC markers. **a** siRNA mediated knockdown of GALNT3 and immunoblotting analysis of CSC markers expression from SP cells of SW1990 and Capan1, respectively. **b** siRNA mediated knockdown of B3GNT3 and immunoblotting analysis of CSC markers expression from SP cells of SW1990 and Capan1, respectively. **c** IP of CD44s and CD44v6 from SP cells of SW1990 and Capan1, and immunoblotting with CD44s, CD44v6 and sLe^a^. **d** IP of sLea from SP cells of SW1990, and immunoblotting with sLe^a^, CD44v6 and CD44s

To identify the mechanism by which GALNT3 and B3GNT3 impact CSC stemness, we performed transient KD of GALNT3 and  B3GNT3 in PCSCs. Silencing of GALNT3 and B3GNT3 showed altered expression of the cell surface, and self-renewal stem cell markers in PCSCs. Specifically, GALNT3 KD reduced expression levels of SOX2, OCT3/4 and ESA (Fig. 7a). KD of B3GNT3 reduced CD44v6, SOX2 and OCT3/4 expression (Fig. 7b). Our results suggest that both GALNT3 and B3GNT3 maintain stemness by altering expression of PCSC markers.

### CD44v6 carries sLe^a^ glycans in PCSCs

In SP cells of SW1990 and Capan1, increased expression of sLe^a^ glycans at higher molecular weight (100-250 kDa) was observed. Since CD44v6 molecular weight falls in that size range, we analyzed for its potential modification with sLe^a^. CD44S and CD44v6 were immunoprecipitated and blotted with sLe^a^ expression in PCSCs. We were interested to find that only CD44v6 carries sLe^a^ modification in significant amounts, but not CD44S in SW1990 and Capan1 SP cells (Fig. 7c). We also performed reciprocal IP with sLe^a^ and probed for CD44v6 and CD44S, and found that CD44v6 underwent sLe^a^ modification (Fig. 7d). Our results indicate that the modification of sLe^a^ on CD44v6 of PCSC, which may be involved in tumor metastasis.

### GALNT3 regulates stemness in PCSCs

**Fig. 8:**
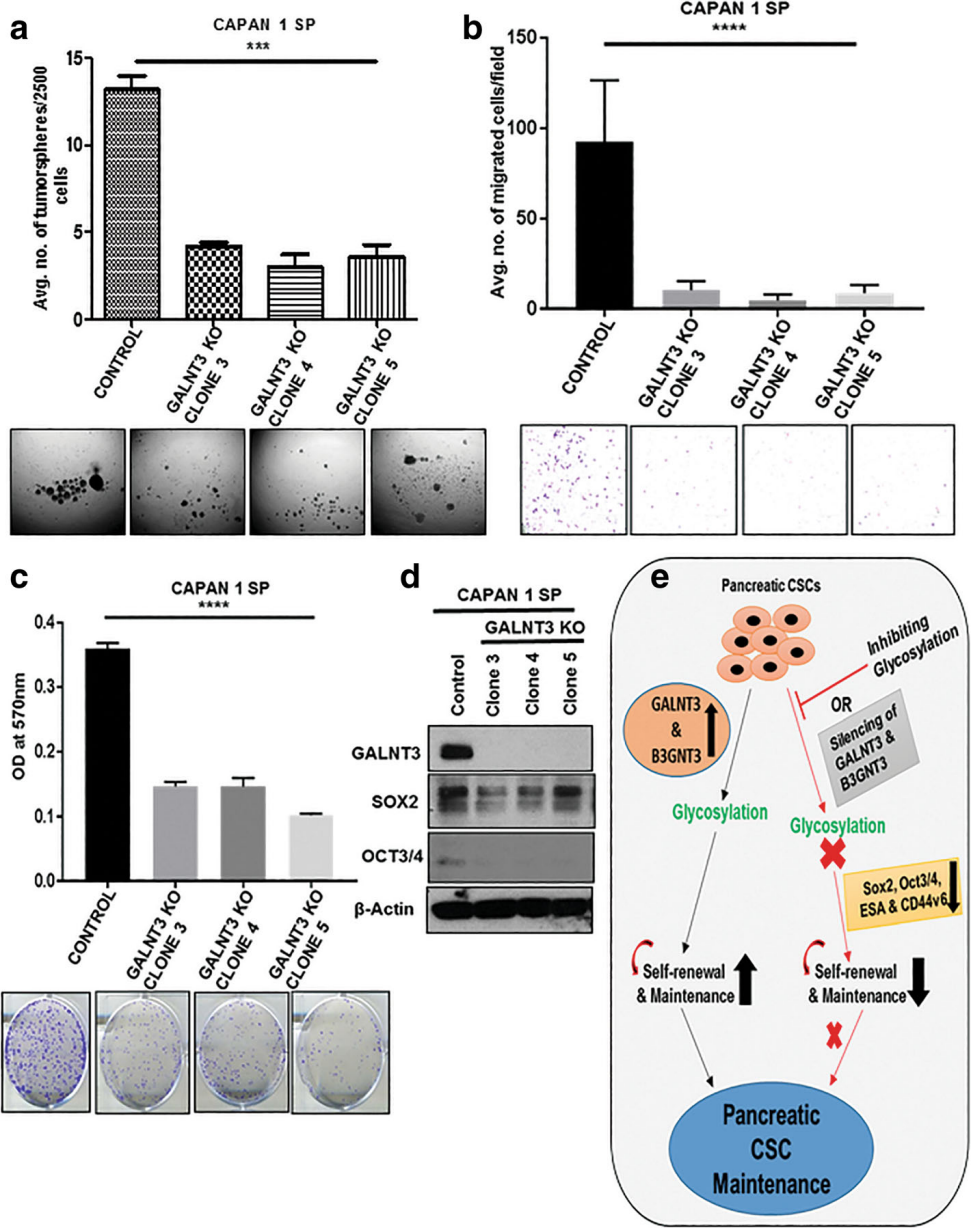
GALNT3 regulates stemness, clonogenicity and migration in PCSCs. **a** Tumorsphere sphere formation assay in GALNT3 KO clones compared to control Capan 1 SP cells. **b** and **c** Transwell migration and colony formation assay in GALNT3 KO clones compared to control SP cells. **d** Immunoblotting analysis of CSC markers expression compared to control SP cells. **e** Schematic representation describing the importance of glycosylation in the maintenance of stemness of PCSCs. Silencing of GALNT3 and B3GNT3 specifically involved in decreasing the self-renewal markers and stemness in PCSCs

Transient KD of GALNT3 alluded to its role in regulating the expression of PCSCs markers. We further used CRISPR/Cas9 system to KO GALNT3 expression to explore its functional importance in PCSCs, with three complete GALNT3 KO Capan1 SP clones developed (Fig. 8d, Additional file 6: Figure S5). GALNT3 KO clones formed a lesser number of tumorspheres compared to control SP cells (Fig. 8a). KO of GALNT3 resulted in loss of self-renewal and the in-vitro tumorigenic potential of SP cells. We next evaluated the effect of GALNT3 KO on clonogenicity and migration of SP cells. GALNT3 KO clones formed very few colonies (Fig. 8c), and migrated much less compared to control SP cells (Fig. 8b). Furthermore, GALNT3 KO clones showed reduced expression of self-renewal markers, such as SOX2 and OCT3/4 (Fig. 8d). These results imply that GALNT3 regulates growth, stemness, and migration of PCSCs.

## Discussion

Cancer stem cells have been correlated to metastasis to distant organs and to chemo- and radio- resistance including pancreatic cancer [11, 36]. Although there is ongoing work to target CSCs, mainly at the genomic and proteomic levels, CSC nevertheless remains a challenge that must be addressed. It is crucial, therefore, to understand CSC maintenance from a unique angle that may pave the way for better management of PC. To that end, in the present study, we investigated the importance of glycosylation in maintenance of PCSC populations, identifying and defining the role of GALNT3 and B3GNT3 in maintenance of stemness of PCSCs.

CSCs or tumor initiating cells (TICs) are responsible for tumor initiation, metastasis, chemo/radio-resistance, and recurrence in many cancers including PC [4, 5, 11, 36]. Aberrant glycosylation is also implicated for tumor development, metastasis and chemoresistance in PC and several other malignancies [15–17]. Our results indicate that glycosylation plays an important role in the maintenance of stemness of PCSCs. Inhibition of *N*- and *O*-linked glycosylation decreased SP/CSCs, in PC cells suggesting that both glycan modifications are important for PCSCs maintenance. Further, inhibition of *N*-linked glycosylation (TM treatment) reduced tumorsphere formation, clonogenicity, migration, and hypo-glycosylated CD44v6 and ESA of PCSCs. TM, a mixture of homologous nucleoside antibiotics, blocks *N*-linked glycosylation by inhibiting GlcNAc phophotransferase. TM has been reported to induce unfolded protein response (UPR) and endoplasmic reticulum (ER)-stress mediated apoptosis in many cancers [37, 38]. Similar to our observation, a previous study shows TM treatment reduces the in vitro subpopulation and invasion of breast CSCs through ER stress [39]. In another study, TM treatment increased hypo-glycosylated ESA by decreasing its glycosylated form in head and neck cancer [35]. Our results with these studies suggest that *N*-linked glycosylation is involved in the maintenance of PCSCs by altering the glycosylation of CSC markers.

Based on the importance of glycosylation in PCSCs, we further identified the glycogenes involved in stemness. Our results indicated upregulation of GALNT3 and B3GNT3 among 84 GFs in isolated CSCs/SP cells. Both GALNT3 and B3GNT3 are involved in the synthesis of mucin-type *O*-linked glycosylation. For example, GALNT3 is member of GalNAc-transferases that catalyze the addition of GalNAc on the serine or threonine residue of polypeptide to from the Tn structure [40]. B3GNT3 is member of the B3GlcNAc family that catalyzes the extended core 1 structure (6-sulfo sLe^x^), synthesizes the poly-N-acetyllactoseamine structures and dimeric sLe^a^ antigens, and is involved in L-selectin ligand biosynthesis, lymphocyte homing, and trafficking [41–43]. Since GALNT3 is linked to biosynthesis of the Tn carbohydrate antigen and B3GNT3 is correlated to the sLe^a^ antigen, increased expression of GALNT3 and B3GNT3 in CSCs could be the plausible mechanism of observed increased expression of Tn and sLe^a^ in PCSCs.

The expression of GALNT3 and B3GNT3 along with CSC marker CD44v6 were analyzed in the progression of KC and KPC mouse tissues. In the KC model, PanIN (Pancreatic Intraepithelial neoplasia) I lesions start at 10 weeks of age and develop to fully blown PDAC (Pancreatic ductal adeno carcinoma) at 50th weeks of age [32]. In KPC model, PanINs observed at 5 weeks of age that progress to PDAC stage by 20th and 25th weeks of age [29]. Our results showed co-expression of GALNT3 and B3GNT3 along with CD44v6 at an advanced stage of PDAC in KC (50th week) and KPC (20th and 25th week). These results suggest the possible role of GALNT3 and B3GNT3 in the maintenance of CSCs in advanced stages of PC progression.

GALNT3 and B3GNT3 have been implicated in the regulation of tumorigenesis in many cancers. GALNT3 is overexpressed in some cancers including PC, and is known for its tumor-promoting role in pancreatic and ovarian cancers [44, 45]. In our previous study, by contrast, we demonstrated that down-regulation of GALNT3 leads to aggressiveness in poorly differentiated PC cells [46]. In the present study, significantly increased expression of GALNT3 was shown in PCSCs, which is an undifferentiated population. These studies and our observation suggest that GALNT3 is differentially expressed based on the differentiation potential of cancer cells, and may play a different functional role based on its level of expression. Similarly, we demonstrated the multifunctional role of PD2/PAF1, a stem cell marker, based on the expressional variation in different cell types [29, 47–51]. Expression of B3GNT3 is also involved in the development and progression of many cancers, including non-hodgkin lymphoma (NHL), colon, pancreatic, esophageal squamous cell, hepatocellular, and cervical [41, 43, 52, 53]. In contrast, over expression of B3GNT3 increases the migration and invasion in neuroblastoma cells [54]. These reports indicate opposite roles of these GFs in the tumorigenesis of different cancers.

Our next goal was to investigate the GALNT3 and B3GNT3 mediated mechanism for maintenance of PCSCs. We observed that KD of GALNT3 reduced expression of SOX2, OCT3/4, and ESA, and that B3GNT3 KD reduced CD44v6, SOX2 and OCT3/4 expression. These results confirm that *O*-GFs GALNT3 and B3GNT3 are involved in the maintenance of stemness in PCSCs by altering the CSC markers. Similar to our observation, numerous studies have shown the significant involvement of GFs in the regulation of stemness of many cancers [25]. GALNT1 has been reported to regulate stemness in bladder CSCs through activating SHH signaling and inducing gli1 expression [55]. The two GFs, B4GALNT3 and MGAT5, are involved in synthesis of *N*-linked glycosylation and have been implicated in regulating stemness in colon CSCs. B4GALNT3 and MGAT5 have been shown to regulate stemness by modifying EGFR and WNT glycosylation, respectively [56, 57]. In another study, ST6Gal-1 has been reported to regulate stemness and gemcitabine resistance in pancreatic and ovarian cancer by modulating SOX9 and SLUG expression [27]. Our study supports the concept that specific GFs are involved in the maintenance of stemness in PC.

Expression of TACAs are implicated in tumorigenesis in numerous cancers [15–17]. Therefore, we analyzed the expression of TACAs in PCSC, given that they are implicated in tumor development and metastasis. Our results showed a global change in expression of TACAs, and increased expression of Tn and sLe^a^ at higher molecular weight proteins. Further, we noticed the reduction in expression of CD44v6 in PCSCs by KD of B3GNT3. Because both CD44v6 and sLe^a^ are implicated in the tumor metastasis process, B3GNT3 may be involved in the possible modification of CD44 for sLe^a^ in PCSCs. Of interest, our IP results showed the modification of sLe^a^ on CD44v6 but not on CD44S in PCSCs. There are many reports in the literature relating the expression of CD44v6 and sLe^a^ to tumor metastasis. CD44v6 expression promotes tumor growth and metastasis in many cancer including PC and colorectal CSCs [58–62]. In one study, sLe^a^ modification on CD44v6 was shown to mediate detachment of ploymorphonuclear leukocytes during trans-epithelial migration from the intestinal epithelium [63]. The H type glycan on CD44v6 was also shown to augment motility of tumor cells and tumorigencity in rat colon carcinoma [64, 65]. Further, CD44v6 is known to be modified with T and sTn antigens in colon cancer and is implicated in tumor metastasis [66]. Our results and those of previous studies indicate the possible involvement of CD44v6 modified with sLe^a^, possibly by B3GNT3, which may facilitate tumor metastasis. However, this insight warrants further detailed investigation.

CRISPR/Cas9-mediated GALNT3 KO in PCSCs showed decreased tumorspheres formation, colony formation and migration potential, along with reduced expression of the stem cell transcription factors, SOX2 and OCT3/4. These results suggest that GALNT3 regulates stemness, growth and migration by regulating SOX2 and OCT3/4 expression in PCSCs. Similar to our observation, stable knockdown of GALNT1 was shown to inhibit oncosphere formation and soft agar colony formation in bladder CSCs by regulating gli1 expression [55]. Our results and the literature further support the involvement of GFs in regulating stemness of CSCs.

## Conclusions

In summary, our present work thus delineates the importance of glycosylation in the maintenance of stemness in PCSCs. We have identified for the first time the role of GALNT3 and B3GNT3 in regulating the stemness of PCSCs by altering CSC markers (Fig. 8e). Specifically, modification of sLe^a^ on CD44v6 occurs in PCSCs, which may be involved in the tumor metastasis. We conclude that *O*-GFs GALNT3 and B3GNT3 are involved in the maintenance of PCSCs and the observed findings opens new avenues to target CSC populations in PC.

## Additional files

Additional file 1: **Table S1.** Antibodies used for the study. **Table S2.** Primers used for the study. A. Cancer stem cell markers and B. Glycogenes. **Table S3.** Fold change values of differentially expressed genes in SP when compared to NSP of SW1990. (DOCX 21 kb)

Additional file 2: **Figure S1**. TM and BAG inhibits growth and alters glycosylation of PC cells. (a) and (b), Effect of TM on growth of SW1990 and Capan1 cells. (c) and (d), Effect of BAG on growth of SW1990 and Capan1 cells. (e) Effect of TM on *N*-linked glycosylation of PC cells. (f) Effect of BAG treatment on *O*-linked glycosylation of PC cells. (TIF 2768 kb)

Additional file 3: **Figure S2.** Isolation and Characterization of PCSCs. Isolation and Characterization of PCSCs. (a) Sorting of SP and NSP cells from SW1990 and Capan 1 by Hoechst staining. Reserpine/Verapamil used as control to gate the SP cells. (b) Morphology of NSP and SP cells of SW1990 and Capan1. (c) and (d), RT-qPCR analysis of CSC markers expression between NSP and SP cells of SW1990 and Capan1, respectively. β-Actin is used to normalize the fold change values. (TIF 4154 kb)

Additional file 4: **Figure S3.** RT-qPCR analysis of DEGs in PCSCs. (a) and (b), RT-qPCR analysis for validation of DEGs identified by PCR array between NSP and SP cells of SW1990 and Capan1. (TIF 2283 kb)

Additional file 5: **Figure S4.** GALNT3 and B3GNT3 expresses at PDAC stage in PCSCs of KC. (a) and (b), IF analysis for co-expression of GALNT3 and B3GNT3 with CD44v6, respectively, at different stages of KC. (TIF 8121 kb)

Additional file 6: **Figure S5.** KO of GALNT3 in Capan 1 SP cells. IF analysis for expression of GALNT3 in control and GALNT3 KO clones of Capan 1 SP. (TIF 2895 kb)

## Abbreviations

BAG: Benzyl-2-acetamido-2-deoxy-α-D-galactopyranoside
CSCs: Cancer stem cells
DEGs: Differentially expressed glycogenes
DMEM: Dulbecco’s Modified Eagles Medium
DMSO: Dimethyl sulfoxide
ER: Endoplasmic reticulum
FACS: Fluorescence activated cell sorting
GFs: Glycosyltransferases
IF: Immunofluorescence
IP: Immunoprecipitation
KC: *Kras* ^*G12D*^ *; Pdx-1-Cre*
KD: Knockdown
KO: Knockout
KPC: *Kras* ^*G12D*^ *; p53* ^*R172H*^ *; Pdx-1-Cre*
NHL: Non-hodgkin lymphoma
NSP: Non side population
PanIN: Pancreatic Intraepithelial neoplasia
PC: Pancreatic cancer
PCR: Polymerase chain reaction
PCSCs: Pancreatic cancer stem cells
PDAC: Pancreatic ductal adenocarcinoma
PVDF: Polyvinylidene difluoride
RT-qPCR: Real time quantitative polymerase chain reaction
SDS-PAGE: Sodium dodecyl sulfate polyacrylamide gel electrophoresis
sLe^a^: Sialyl Lewis a
sLe^x^: Sialyl Lewis x
SP: Side population
TACAs: Tumor associated carbohydrate antigens
TICs: Tumor initiating cells
TM: Tunicamycin
UPR: Unfolded protein response
