# Supplementary material for: Novel role of O-glycosyltransferases GALNT3 and B3GNT3 in the self-renewal of pancreatic cancer stem cells
Source: BMC Cancer. 2018 Nov 22;18:1157. doi: 10.1186/s12885-018-5074-2 (PMC6251200; doi:10.1186/s12885-018-5074-2)
Supplement: Supplementary file 1 — Table S1. Antibodies used for the study. Table S2. Primers used for the study. A. Cancer stem cell markers and B. Glycogenes. Table S3. Fold change values of differentially expressed genes in SP when compared to NSP of SW1990. (DOCX 21 kb) [file 12885_2018_5074_MOESM1_ESM.docx]

# **Additional file 1**

**Table S1: Antibodies used for the study**

| **Antibody** | **Company** |
| --- | --- |
| CC49 | (Sheer et al., 1988) |
| SLe^x^ | (Ogata et al., 1995) |

**References:**

Ogata S, Ho I, Chen A, Dubois D, Maklansky J, Singhal A, Hakomori S, Itzkowitz SH (1995) Tumor-associated sialylated antigens are constitutively expressed in normal human colonic mucosa. Cancer Res 55 (9): 1869-1874.

Sheer DG, Schlom J, Cooper HL (1988) Purification and composition of the human tumor- associated glycoprotein (TAG-72) defined by monoclonal antibodies CC49 and B72.3. Cancer Res 48 (23): 6811-6818.

**Table S2: Primers used for the study**

**A. Cancer stem cell markers**

| **Sl. No.** | **Gene** | **Forward Primers (5′ → 3′)**  **Reverse Primers (5′ → 3′)** | **Size (bp)** |
| --- | --- | --- | --- |
| 1 | CD44S | GGAGCAGCACTTCAGGAGGTTAC  GGAATGTGTCTTGGTCTCTGGTAGC | 129 |
| 2 | CD44v6 | CCAGGCAACTCCTAGTAGTACAACG  CGAATGGGAGTCTTCTTTGGGT | 112 |
| 3 | ALDH1A3 | ATCAACTGCTACAACGCCCT  TATTCGGCCAAAGCGTATTC | 98 |
| 4 | ABCG2 | GAGCCTACAACTGGCTTAGACTCAA  TGATTGTTCGTCCCTGCTTAGAC | 85 |

**B. Glycogenes**

| **Sl. No.** | **Gene** | **Forward Primers (5′ → 3′)**  **Reverse Primers (5′ → 3′)** | **Size (bp)** |
| --- | --- | --- | --- |
| 1 | B3GNT2 | GGCCTCTACCCACCCTATGC AGGCCGAGTTTCTGAAGGCA | 143 |
| 2 | B3GNT3 | GCAACGCCTGTCCTCCTTTG  GATTGCCGCAGGTGAGGTTG | 140 |
| 3 | GALNT12 | AGCCTGGTCAACTCTCCTTCG  GGGCAGTCCCGAAAGCTCAT | 145 |
| 4 | GALNT16 | TGTGGATGTGTGGTGGCAGT  CACACTTCTGCAGTGCGCTT | 139 |
| 5 | FUCA1 | CACCTGCGCGTTAAGAGTGG  GGAAGAGCAGCAGCAGCAAC | 106 |
| 6 | FUT8 | CTGGGTCCCAAGGCTACAGG CCGCCGAGGCCAATCAGA | 114 |
| 7 | GCNT2 (C2GnT-M) | GCCTTGTGAAGAGATCATCC  CAGAATAGCCTGAAGCACTG | 364 |
| 8 | GCNT3  (C2GnT3) | CCTGGCTCTGTTCCCAACCA  CAGATAGCCCGCTGGTGGAT | 146 |
| 9 | MAN1A1 | CCTGTTGCCGCTCTTCAGC AGCAGCACGAACTTCTCCGT | 120 |
| 10 | MAN2A1 | CGCTGAGCTTGCGATCAAGT  TTGCTCCGCACCTAGTCTCC | 149 |
| 11 | MGAT4A | GTGTGTGCTGTCTCTCTGACCA CCCTGAGCACCCACCATAGG | 94 |
| 12 | MGAT4B | ACTTCATCCGCTTCCGCTTC  TCCTTGTCTGACTGAGGGTTGT |  |
| 13 | OGT | AGCTCCAGATGGCGTCTTCC  GCAGTGTCTCTCAGCTGCCT | 110 |
| 14 | POMT2 | GACCATCCGGCCATGAGGAA  GGCTCTCTCCAATGCTGGGT | 109 |
| 15 | ST6GAL1 | TAGCAAGTGCAGCCTCACGA  AACCCAGCTGACAGGACAGG | 100 |
| 16 | ST6GALNAc1 | TGGAGATGCAGGCACCTGAG GTGCGTTGATGCCTGGAAGG | 128 |
| 17 | HPRT1 | ACCCGCAGCCCTGGCGTCGT ACACCCTTTCCAAATCCTCAGCA | 148 |

**Table S3: Fold change values of differentially expressed genes in SP when compared to NSP of SW1990**

| **Sl.**  **No.** | **Position** | **Gene Symbol** | **Sugar** | **PCR Array**  **Fold Change** | **RT-qPCR**  **Fold Change** |
| --- | --- | --- | --- | --- | --- |
| **1** | A04 | B3GNT2 | GlcNAc | 2.39 | 1.6 |
| **2** | A05 | B3GNT3 | GlcNAc | 7.78 | 7.1 |
| **3** | A12 | C1GALT1 | Gal | 2.99 | 0.73 |
| **4** | B05 | FUCA1 | Fucose (Fucosidase) | 2.81 | 0.85 |
| **5** | B08 | FUT8 | Fucose | 2.97 | 0.59 |
| **6** | B09 | GALNT1 | GalNAc | 2.33 | 1.33 |
| **7** | B12 | GALNT12 | GalNAc | 7.73 | 5.42 |
| **8** | C02 | GALNT14 | GalNAc | 4.29 | 0.73 |
| **9** | C04 | GALNT3 | GalNAc | 12.38 | 12.99 |
| **10** | C06 | GALNT6 | GalNAc | 2.19 | 0.97 |
| **11** | C10 | GALNT16 | GalNAc | -2.23 | 0.22 |
| **12** | D03 | GCNT3 | GlcNAc | 7.06 | 0.67 |
| **13** | D10 | MAN1A1 | Manose (Mannosidase) | -2.07 | 0.85 |
| **14** | E02 | MAN2A1 | Manose (Mannosidase) | 2.06 | 0.81 |
| **15** | E09 | MGAT4A | GlcNAc | 4.63 | 2.14 |
| **16** | F08 | OGT | GlcNAc | 3.78 | 2.14 |
| **17** | G01 | POMT2 | Mannose | 2.25 | 0.28 |
| **18** | G05 | ST6GAL1 | NeuAc | -3.25 | 0.23 |
| **19** | G06 | ST6GALNAC1 | NeuAc | 2.77 | 0.73 |
| **20** | H04 | HPRT | Ribose | 2.91 | 0.67 |
