# Supplementary figures and images for: Novel role of O-glycosyltransferases GALNT3 and B3GNT3 in the self-renewal of pancreatic cancer stem cells
Source: BMC Cancer. 2018 Nov 22;18:1157. doi: 10.1186/s12885-018-5074-2 (PMC6251200; doi:10.1186/s12885-018-5074-2)

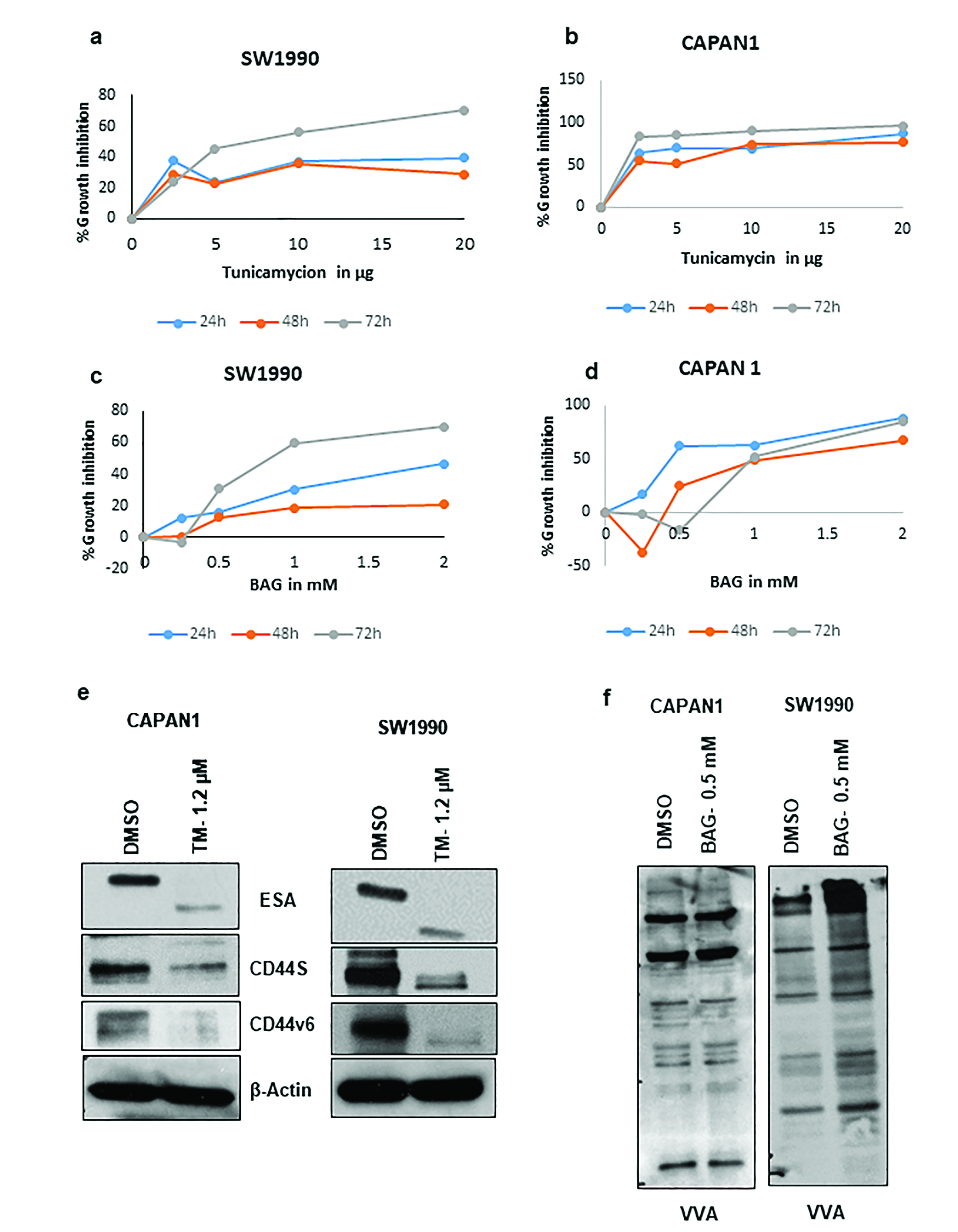

Supplement: Supplementary file 2 — Figure S1. TM and BAG inhibits growth and alters glycosylation of PC cells. (a) and (b), Effect of TM on growth of SW1990 and Capan1 cells. (c) and (d), Effect of BAG on growth of SW1990 and Capan1 cells. (e) Effect of TM on N-linked glycosylation of PC cells. (f) Effect of BAG treatment on O-linked glycosylation of PC cells. (TIF 2768 kb) [file 12885_2018_5074_MOESM2_ESM.tif]

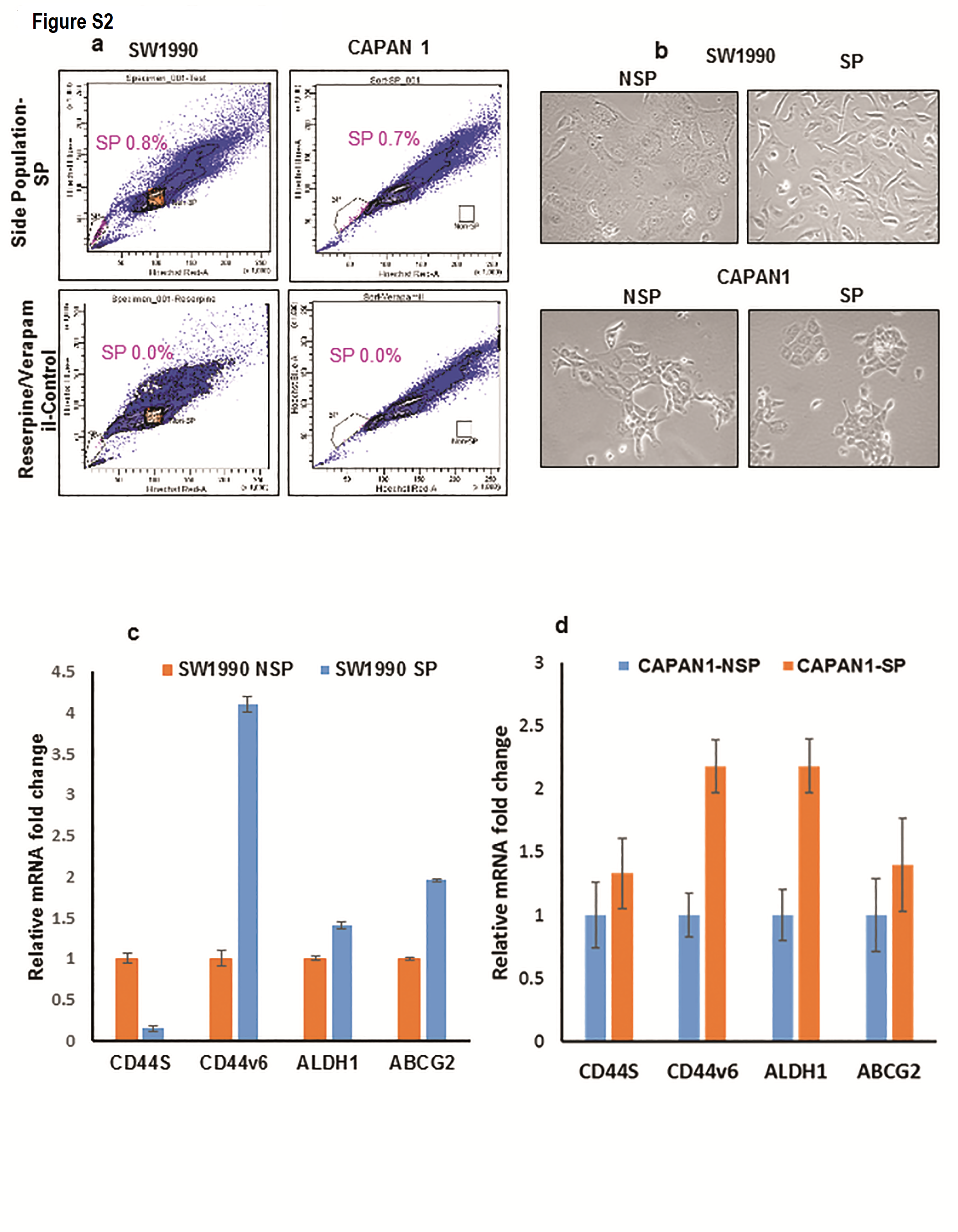

Supplement: Supplementary file 3 — Figure S2. Isolation and Characterization of PCSCs. Isolation and Characterization of PCSCs. (a) Sorting of SP and NSP cells from SW1990 and Capan 1 by Hoechst staining. Reserpine/Verapamil used as control to gate the SP cells. (b) Morphology of NSP and SP cells of SW1990 and Capan1. (c) and (d), RT-qPCR analysis of CSC markers expression between NSP and SP cells of SW1990 and Capan1, respectively. β-Actin is used to normalize the fold change values. (TIF 4154 kb) [file 12885_2018_5074_MOESM3_ESM.tif]

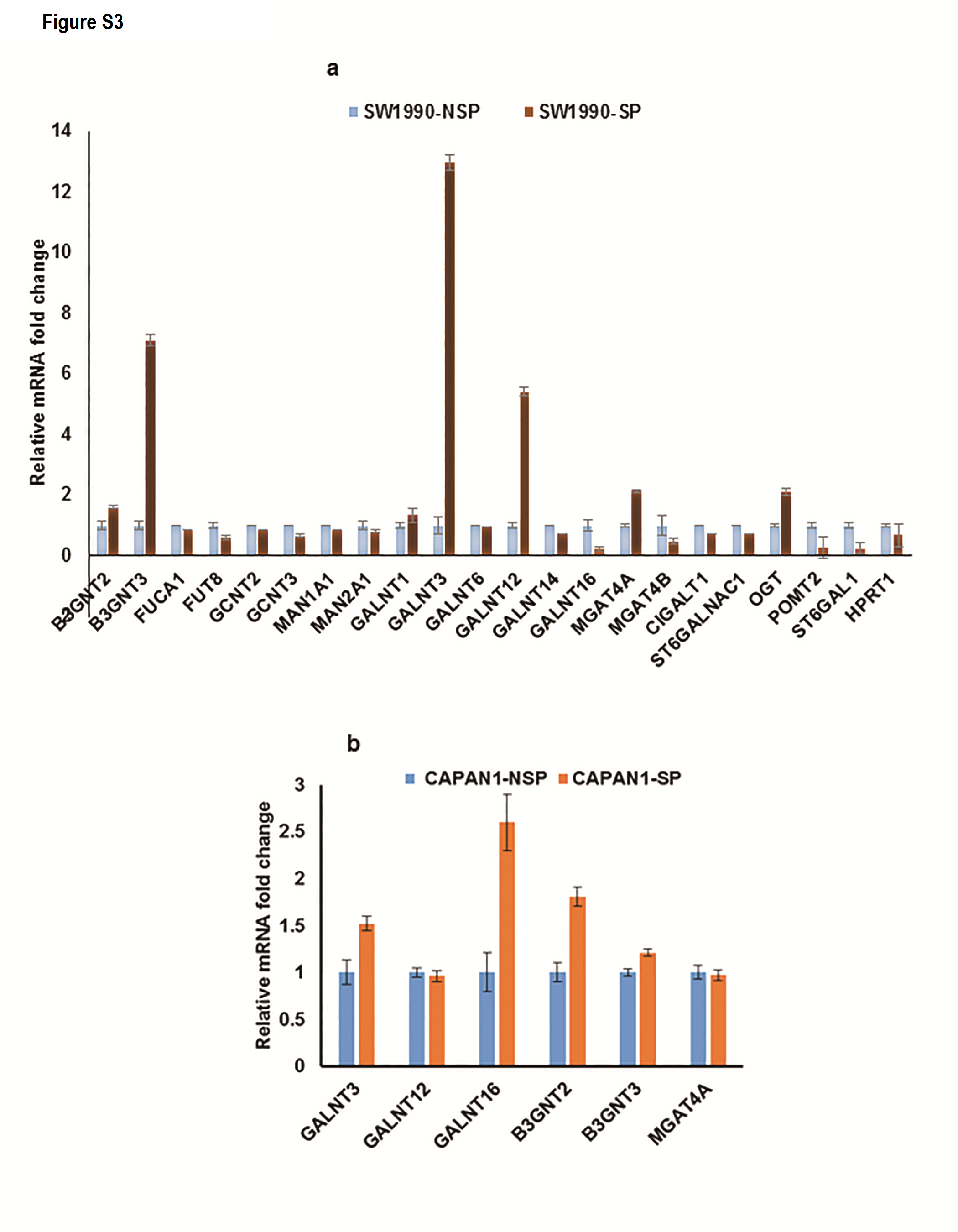

Supplement: Supplementary file 4 — Figure S3. RT-qPCR analysis of DEGs in PCSCs. (a) and (b), RT-qPCR analysis for validation of DEGs identified by PCR array between NSP and SP cells of SW1990 and Capan1. (TIF 2283 kb) [file 12885_2018_5074_MOESM4_ESM.tif]

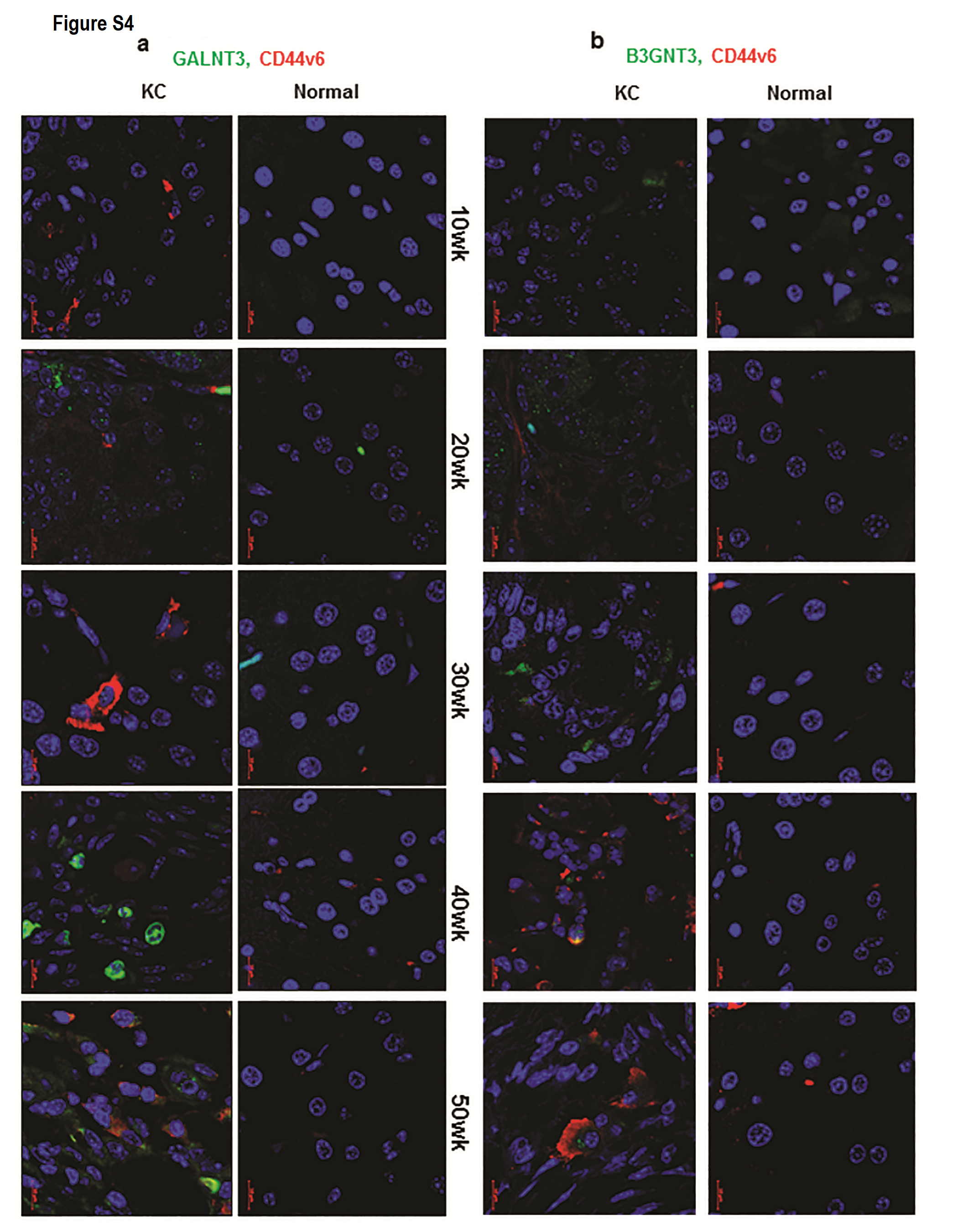

Supplement: Supplementary file 5 — Figure S4. GALNT3 and B3GNT3 expresses at PDAC stage in PCSCs of KC. (a) and (b), IF analysis for co-expression of GALNT3 and B3GNT3 with CD44v6, respectively, at different stages of KC. (TIF 8121 kb) [file 12885_2018_5074_MOESM5_ESM.tif]

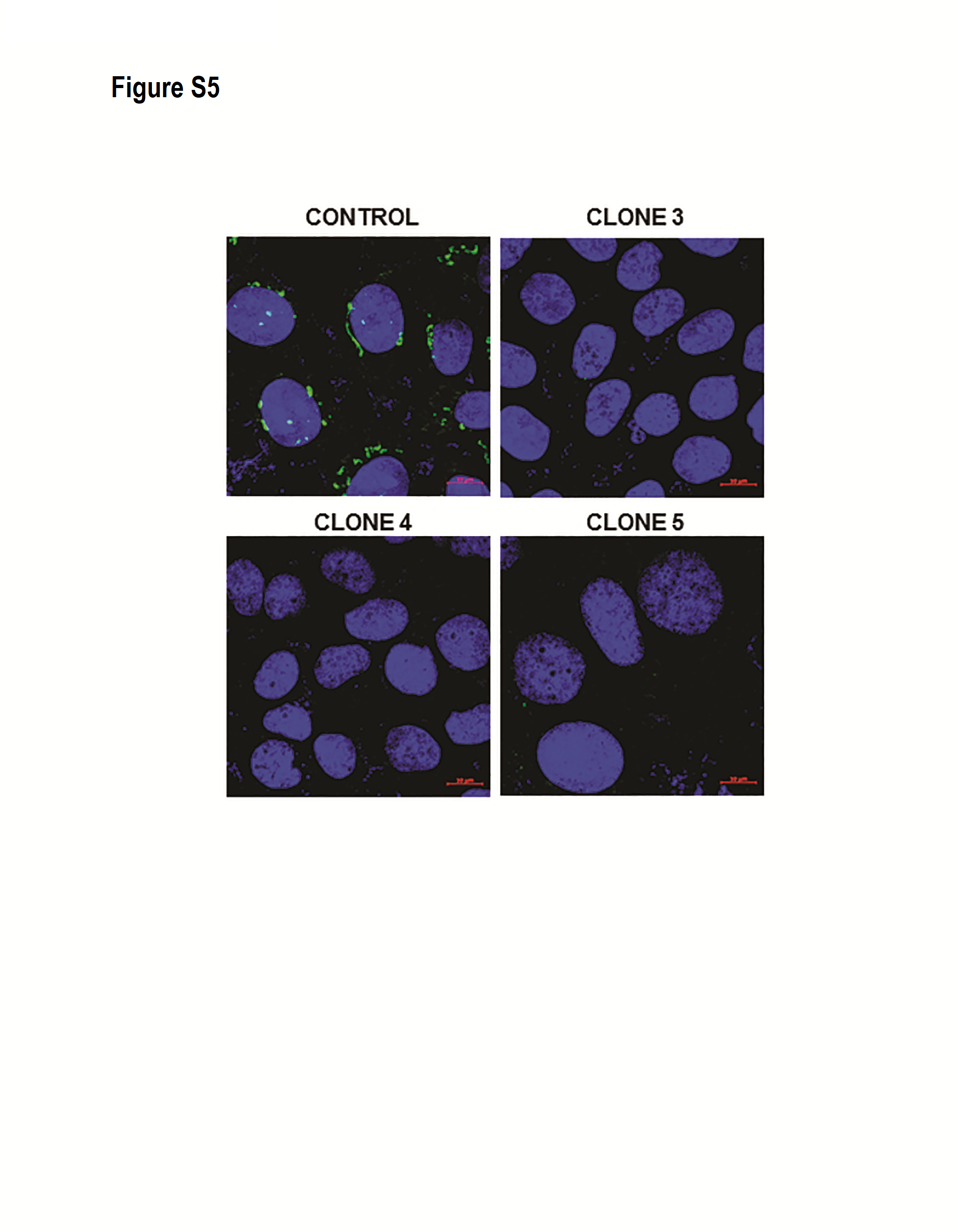

Supplement: Supplementary file 6 — Figure S5. KO of GALNT3 in Capan 1 SP cells. IF analysis for expression of GALNT3 in control and GALNT3 KO clones of Capan 1 SP. (TIF 2895 kb) [file 12885_2018_5074_MOESM6_ESM.tif]
